# Imaging Advances in Light Chain Amyloidosis

**DOI:** 10.3390/diagnostics16142225

**Published:** 2026-07-16

**Authors:** Miaoling Qiu, Kaini Shen, Hua Yang, Jun Wang, Jian Li

**Affiliations:** 1 Department of Hematology, The University of Hong Kong-Shenzhen Hospital, Shenzhen 518053, China; qiuml@hku-szh.org (M.Q.); yangh@hku-szh.org (H.Y.); wangj5@hku-szh.org (J.W.); 2 Shenzhen Clinical Research Center for Rare Diseases, Shenzhen 518053, China; 3 Department of Hematology, State Key Laboratory of Complex Severe and Rare Diseases, Peking Union Medical College Hospital, Chinese Academy of Medical Sciences and Peking Union Medical College, Beijing 100730, China; shenkaini3@sina.com

**Keywords:** light chain amyloidosis, cardiac amyloidosis, echocardiography, magnetic resonance imaging, nuclear medicine imaging, multimodal imaging, integrating artificial intelligence

## Abstract

Light chain (AL) amyloidosis is a systemic disorder caused by plasma cell dyscrasia, with cardiac involvement being the primary determinant of prognosis. Survival outcomes vary significantly across disease stages. This heterogeneity underscores a critical need for early diagnosis, precise risk stratification, and response-adapted therapy. In this context, multimodality imaging has emerged as an indispensable non-invasive tool, providing crucial insights for clinical decision-making. This review synthesizes recent advances in the application of key imaging modalities—echocardiography, magnetic resonance imaging, and nuclear medicine imaging—for evaluating AL amyloidosis. We highlight how these techniques have shifted the paradigm from anatomical assessment to quantitative, multiparametric tissue characterization, ultimately guiding personalized patient management.

## 1. Introduction

Light chain (AL) amyloidosis is a systemic disorder caused by the deposition of misfolded immunoglobulin light chains, secreted by clonal plasma cells, as insoluble fibrils in the extracellular matrix of various organs. This process disrupts normal tissue architecture and function through mass effects and direct cellular toxicity, leading to progressive organ failure [1,2,3,4].

The prognosis of AL amyloidosis is primarily determined by the extent of cardiac involvement, which remains the leading cause of death. Historically, the Mayo 2004 staging system—based on N-terminal pro-brain natriuretic peptide (NT-proBNP) and cardiac troponin T—has effectively stratified prognosis [5]. Patients diagnosed at Mayo Stage IIIB continue to have a poor prognosis, with a median overall survival (OS) of 6 months to 2 years and a persistently high early death rate (20–40% within 1–3 months) [6,7], underscoring the unmet need for earlier and more accurate diagnosis [8]. Outcomes have improved because of effective disease-modifying therapies, particularly daratumumab-based regimens in patients with Mayo Stage I-IIIA disease. These therapeutic advances attenuate prognostic differences between lower-risk groups and increase the need for refined risk stratification. However, even among patients achieving deep hematologic remission, the cardiac organ response rate remains below 50% [9]. This disparity highlights the necessity for more sensitive tools to monitor treatment efficacy and guide timely therapeutic adjustments. Furthermore, the ongoing development of anti-amyloid therapy demands more precise methods for assessing organ response.

Although imaging in cardiac amyloidosis has been extensively reviewed, few recent syntheses have specifically examined how multimodality imaging can address the evolving clinical needs of AL amyloidosis in the contemporary treatment era. This review therefore focuses on the integrated use of advanced echocardiography, cardiac magnetic resonance (CMR), and emerging nuclear imaging tracers. Rather than considering these modalities solely as diagnostic adjuncts, we highlight their expanding roles in refining risk stratification, reconciling hematologic response with organ-level outcomes, and assessing novel disease-specific therapeutic strategies. We also emphasize the critical distinction between imaging-based suspicion and a definitive diagnosis of AL amyloidosis—the latter of which mandates concurrent hematologic evaluation, histopathological confirmation, and subtyping via amyloid typing.

For this narrative review, we searched PubMed for English-language articles published up to May 2026 using combinations of the terms “light chain amyloidosis,” “AL amyloidosis,” “cardiac amyloidosis,” “echocardiography,” “cardiac magnetic resonance,” “positron emission tomography,” “amyloid PET,” “fibroblast activation protein inhibitor,” and “artificial intelligence.” Priority was given to clinical studies; multicenter cohorts; systematic reviews or meta-analyses; consensus statements; and recent studies addressing diagnosis, prognostic stratification, or treatment response monitoring. Preclinical studies, small single-center studies, and early artificial intelligence (AI)/radiomics studies were selectively included when they illustrated emerging research directions not yet ready for routine practice.

## 2. Echocardiography

Echocardiography is the most widely used and cost-effective imaging modality for initial evaluation in patients with cardiac symptoms or suspected cardiac amyloidosis (CA). It provides a comprehensive evaluation of cardiac structure and systolic and diastolic function. Beyond conventional parameters, advanced speckle-tracking echocardiography (STE) offers a more sensitive, quantitative analysis of myocardial deformation. This technique enables precise measurement of the global longitudinal strain (GLS) and myocardial work.

### 2.1. Diagnosis

The characteristic echocardiographic features of CA—including increased LV wall thickness, restrictive diastolic dysfunction, bi-atrial enlargement, and the “5-5-5” sign on tissue Doppler—raise diagnostic suspicion but lack sufficient specificity individually, necessitating integration with low-voltage electrocardiography (ECG) and emerging methods such as inferior vena cava assessment to exclude phenotypically similar conditions such as hypertrophic cardiomyopathy (HCM) or hypertensive heart disease (HHD) [10,11,12].

The aforementioned features appear relatively late in the disease course, whereas STE holds advantages for early diagnosis. CA is characterized by reduced GLS with relative apical sparing (RAS, apical/basal strain ratio >1.0) [13,14], though the diagnostic accuracy of this pattern alone is modest (sensitivity 72%, specificity 66%) [15]. Strain impairment is systemic, involving all cardiac chambers, with right ventricular (RV) and left atrial (LA) strain potentially offering superior diagnostic discrimination [16,17]. Three-dimensional STE (3DSTE) allows for comprehensive assessments of deformation, including torsion [18,19,20]. Early-stage CA shows preserved LV torsion and a high torsion/GLS index. This compensation initially manifests as heart failure with preserved ejection fraction (HFpEF) but ultimately fails with disease progression [19].

Although echocardiography cannot definitively subtype amyloidosis, it reveals suggestive patterns. AL-CA more commonly presents with symmetric hypertrophy and pericardial effusion, while ATTR-CA frequently shows asymmetric hypertrophy, more pronounced apical sparing, and a higher incidence of concomitant aortic stenosis [21,22,23,24]. For a comparable amyloid burden, AL-CA is associated with more severely impaired GLS and lower myocardial work efficiency [25,26], supporting the concept of direct light chain cardiotoxicity.

Given the limitation of any single parameter, integrated multiparametric approaches hold significant promise for substantially enhancing diagnostic performance. Integrated diagnostic approaches that combine conventional parameters and strain-derived ratios (e.g., EF–strain and mass–strain ratios) improve diagnostic performance [27,28,29,30].

Several multiparametric echocardiographic scores have been developed to improve the diagnostic accuracy for CA. The AL score, derived from a large multicenter study, integrates four parameters: relative wall thickness (RWT) > 0.52 (2 points), mitral E/e’ ratio > 10 (2 points), tricuspid annular plane systolic excursion (TAPSE) ≤ 19 mm (1 point), and GLS ≥ −14% (1 point). An AL score of ≥5 points is highly specific (98%) for diagnosing AL-CA, while a score of <1 point effectively rules it out [31]. As a simplified screening tool, the AMYLI score is defined as the product of the RWT and the mitral E/e’ ratio. An AMYLI value below 2.36 effectively excludes AL-CA [32]. A novel Echocardiographic Nomogram Model (using the RWT, E/e’, ejection fraction/peak systolic global longitudinal strain ratio (EFSR), and right ventricular fractional area change (RV FAC)) also offers effective, non-invasive screening for AL-CA with good sensitivity (80.4%) and specificity (85.7%), aiding early detection and reducing the need for biopsy [33]. This model, however, has notable limitations: it not only omits the key parameter of left ventricular strain but also was derived from a cohort of predominantly established CA cases, potentially limiting its generalizability to early-stage disease. Furthermore, it currently lacks external validation.

The integration of echocardiographic data with artificial intelligence (AI) is promising but remains an emerging research area. AI models analyzing combined ECG-echocardiogram data or single echocardiographic videos demonstrate high diagnostic accuracy for CA. They outperform IWT scores and even expert clinicians in differentiating phenocopies and hold potential for early detection [33,34,35]. These results should be interpreted cautiously because many models are retrospective, single-center, and vulnerable to scanner/protocol heterogeneity and limited external validation. At present, AI should be viewed as a decision support tool rather than a replacement for expert interpretation or confirmatory hematologic and histopathological evaluation.

### 2.2. Prognosis

Numerous echocardiographic studies have investigated prognostic assessment in AL amyloidosis, encompassing parameters related to cardiac structure, diastolic and systolic function, myocardial strain, and myocardial work.

LA enlargement and dysfunction are robust prognostic markers in AL amyloidosis. A composite score comprising LA volume and LV-GLS predicts mortality as effectively as the Mayo staging system [36]. An LA volume index of ≥18 mL/m^2^ and left atrioventricular coupling index (LACI) of >0.57 also independently stratify mortality risk [37,38]. RV–pulmonary arterial (PA) coupling, measured by the TAPSE/PASP ratio, provides additional prognostic value. A lower ratio correlates with poorer outcomes, though standardized cutoffs require prospective validation [39,40,41]. Integrated echocardiographic scores further enhance risk prediction. The AMYLI score (≥7.8) independently predicts mortality and improves prognostic performance when combined with clinical data [42].

Deformation imaging, particularly of the GLS, provides highly sensitive prognostic information in AL-CA. To refine risk stratification, a recent international multicenter study integrated GLS with NT-proBNP and high-sensitivity troponin T to create the AL International Staging system (AL-ISS), an enhancement of the Mayo 2004 classification [43]. This system dichotomizes Stage IIIB patients based on GLS into IIIB (GLS < −9%) and IIIC (GLS ≥ −9%), with both sharing stringent biomarker thresholds (NT-proBNP ≥ 8500 ng/L + hs-TnT ≥ 50 ng/L). The study revealed a stark prognostic distinction: Stage IIIC patients had a median survival of only 7 months, compared with 26 months in Stage IIIB patients, while the median survivals for Stages I/II and IIIA were not reached and 67 months, respectively. This GLS-enhanced staging remains prognostically robust in the daratumumab era, clearly identifying Stage IIIC as an ultra-high-risk group.

Beyond LV GLS, impairment of strain in all four cardiac chambers carries prognostic relevance [18,37,44,45]. Among these, LA strain has demonstrated the strongest independent association with OS in comparative analyses [46,47]. Furthermore, myocardial work (MW)—which integrates LV strain with afterload and includes parameters such as the global myocardial work index (GWI) and global constructive work (GCW)—has emerged as a promising indicator. A small retrospective study indicated that GCW is associated with prognosis in AL amyloidosis [48]. A larger prospective study of 96 patients confirmed the GWI and GCW as independent predictors of short-term mortality in AL-CA [49].

Mitral annular plane systolic excursion (MAPSE) reflects left ventricular longitudinal function. Because amyloid infiltration targets subendocardial longitudinal fibers first, MAPSE reduction often precedes LVEF decline, facilitating early risk stratification [50,51]. Nonetheless, GLS offers superior sensitivity and reproducibility. Consequently, MAPSE functions best as a simple, reproducible screening tool and a supplementary index to GLS, rather than a primary diagnostic criterion.

### 2.3. Monitoring Therapy Response

GLS is the most robust and extensively validated echocardiographic parameter for serial assessment of therapeutic response in AL amyloidosis. A large retrospective study from the UK National Amyloidosis Center analyzed 915 patients treated with bortezomib. After a median follow-up period of 50 months, key relationships were established. The data showed that GLS progressively worsened with advancing Mayo 2004 disease stage. Furthermore, this deterioration in GLS was strongly associated with declining OS. Critically, GLS improved significantly at 12 months in patients achieving a complete hematologic response. Patients with a ≥2.0% improvement in their GLS (ΔGLS) at 12 months had superior OS compared with non-responders. Notably, the combination of “GLS improvement” and an “NT-proBNP-based cardiac response” identified a subgroup with significantly longer survival than those achieving a cardiac response by NT-proBNP criteria alone [52]. This prognostic value of GLS improvement was corroborated by a separate study of 97 patients from Memorial Sloan Kettering Cancer Center [53].

A significant challenge for clinical application is the lack of a standardized definition of clinically meaningful GLS improvement. Proposed cutoffs vary, with one study suggesting a value as low as 0.8% [54], highlighting that the optimal threshold and the ideal timing for post-treatment assessment remain unclear. Future large-scale, prospective multicenter studies are imperative to validate these findings, establish consensus thresholds for GLS response, and define the optimal monitoring schedule to guide clinical management.

Accurate interpretation of treatment response necessitates clear differentiation between hematologic response and cardiac organ response. While hematologic response denotes suppression of the underlying clonal plasma cell population, cardiac organ response is conventionally defined by reductions in NT-proBNP and improvements in functional status. Although imaging biomarkers such as GLS offer a more direct assessment of myocardial recovery, they remain distinct from and non-interchangeable with hematologic criteria. Furthermore, while NT-proBNP is a clinical cornerstone, its reliability can be confounded by renal impairment, atrial arrhythmias, volume fluctuations, and treatment-induced fluid shifts. Consequently, the serial integration of imaging with biomarkers—interpreted within the broader clinical context—provides the most robust framework for monitoring therapeutic efficacy.

### 2.4. Key Points

While conventional echocardiography identifies characteristic structural and functional abnormalities in CA, GLS provides the highest diagnostic specificity and is valuable for early detection. GLS extends prognostic value beyond biomarkers to refine risk stratification and serves as the most robust echocardiographic parameter for monitoring treatment response. Standardizing its application requires future prospective studies to define protocols, thresholds, and timing.

## 3. Magnetic Resonance Imaging (MRI)

**Figure 1:**
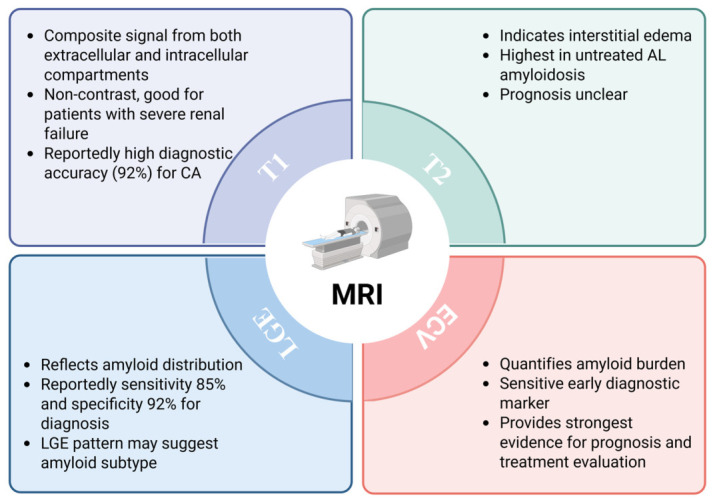
Application of magnetic resonance imaging and key parameters in AL amyloidosis. CA, cardiac amyloidosis; AL, light chain; LGE, late gadolinium enhancement. Created in BioRender. Qiu, M. (2026). https://BioRender.com/5nrmmye (accessed on 26 May 2026).

MRI, especially cardiac magnetic resonance (CMR), has emerged as a powerful tool in the evaluation of cardiac amyloidosis, owing to its superior tissue characterization, high spatial resolution, and comprehensive quantitative capabilities. It provides a wide range of structural, functional, and tissue-specific parameters crucial for diagnosis, prognosis, and response monitoring (Figure 1). Its application is contraindicated in patients with certain metallic implants or severe renal failure.

### 3.1. Diagnosis

Characteristic CMR features include a pattern of global subendocardial or transmural late gadolinium enhancement (LGE) with abnormal gadolinium kinetics, elevated native T1 and T2 values, and expansion of the extracellular volume fraction (ECV).

Cardiac amyloidosis is characterized by abnormal gadolinium kinetics. The expansion of the interstitial space by amyloid deposits alters myocardial contrast dynamics, frequently causing the myocardium to null contemporaneously with or earlier than the blood pool. This complicates the determination of the appropriate inversion time for LGE and precludes the reliance on standard LGE protocols alone [55]. However, recognizing this kinetic pattern—especially when it is integrated with quantitative parameters such as the ECV and native T1, along with clinical and biomarker data—is crucial for diagnosing this diffuse infiltrative process.

The characteristic global subendocardial or transmural LGE pattern—reflecting the amyloid distribution—achieves 85% sensitivity and 92% specificity in differentiating CA from hypertrophic cardiomyopathy [55,56]. The LGE pattern may suggest the amyloid subtype, with a global subendocardial pattern favoring AL-CA and a transmural pattern suggesting ATTR-CA [57]. Atrial late gadolinium enhancement (LGE) is a CMR marker of atrial involvement in cardiac amyloidosis. It is more extensive in cardiac amyloidosis than in HHD or nonischemic dilated cardiomyopathy, and it correlates with impaired left atrial function. However, current evidence supports its use only as a complementary CMR marker [58].

Native myocardial T1—using non-contrast CMR—reflects a composite signal from the extracellular and intracellular compartments, which is significantly elevated in CA. Advanced techniques such as T1ρ and the derived myocardial diffusion index (MDI) may offer superior diagnostic performance in distinguishing CA from HCM compared with the ECV and native T1, though they require further validation [59].

As amyloid deposits expand the extracellular matrix, the ECV—derived from contrast-enhanced T1 mapping—serves as a direct quantitative marker of amyloid burden [60]. It is a sensitive early diagnostic marker, often abnormal before LGE becomes positive [61,62,63]. An ECV of >0.4 is recognized by expert consensus as being indicative of CA [14]. Furthermore, ECV mapping extends beyond cardiac assessment to quantitatively evaluate involvement in extracardiac organs such as the spleen and liver [64,65].

An elevated T2 value indicates interstitial edema, being highest in untreated AL-CA compared with treated AL-CA and ATTR-CA [66]. Combining T2 mapping with LGE patterns can improve accuracy in distinguishing AL-CA from ATTR-CA [67].

A stress perfusion CMR study uncovered profound microvascular dysfunction in CA. The study found that the reduction in myocardial blood flow (MBF) was more severe than that in triple-vessel coronary disease and correlated with the extent of amyloid infiltration [68].

The integration of artificial intelligence (AI) with CMR data shows promise in automating diagnosis and subtyping. For instance, a deep learning model using LGE and cine images achieved AUCs of >0.95 for CA detection [69]. Similarly, a machine learning cascade model accurately distinguished between different CA subtypes, with AUC values ranging from 0.92 to 1.0 [70]. Radiomics approaches also offer substantial diagnostic value, though they require further validation before routine clinical implementation [71,72].

### 3.2. Prognosis

CMR provides powerful prognostic biomarkers for AL amyloidosis, with the ECV holding the most robust evidence. A prospective study demonstrated that incorporating the ECV into the Mayo staging system significantly improved risk prediction, with an ECV of >48% indicating a very high risk [73]. Multicenter data confirm an ECV of ≥45% as an independent prognostic predictor, outperforming established biomarkers such as NT-proBNP and troponin I [74,75]. The central prognostic role of the ECV has been consolidated by meta-analyses [76,77].

Beyond the ECV, several other parameters show prognostic promise, though they require further validation. Elevated myocardial T2 values have been suggested to independently predict outcomes [66], though a meta-analysis indicates that the prognostic significance of native T2 remains uncertain [77]. Reduced myocardial perfusion reserve is associated with prognosis and may improve risk stratification when combined with the ECV in advanced disease [78]. The LACI serves as an independent prognostic factor, adding incremental value in advanced AL amyloidosis [79]. RV longitudinal strain also predicts all-cause mortality [44], and parameters such as LA function and torsion mechanics show associative trends [80,81]. Notably, the current evidence for these advanced markers is largely derived from single-center, retrospective studies with limited sample sizes, underscoring the need for validation in larger, prospective, multicenter studies.

AI applied to CMR data offers a novel approach to prognostication. A single-center retrospective study developed a fully automated deep learning model based on LGE images that accurately predicted individualized prognosis in AL-CA. It effectively distinguished patients with differing outcomes even when they were in the same Mayo stage. This deep learning model outperformed the traditional Mayo staging system [82]. Similarly, a multicenter retrospective study demonstrated that radiomics features extracted from LGE images (particularly a radiomic signature from the LV basal segment) significantly outperformed conventional quantitative LGE parameters in predicting all-cause mortality in CA. They also enhanced the predictive efficacy of the Mayo staging system [83]. Although these results are encouraging, future validation must address external generalizability, feature transparency, segmentation reproducibility, and cross-scanner calibration before broad clinical adoption.

### 3.3. Monitoring Therapy Response

CMR is increasingly used to monitor treatment response in AL amyloidosis, with the ECV providing the strongest evidence base. An ECV reduction reflects a decrease in the interstitial amyloid burden. A prospective study from the UK National Amyloidosis Centre demonstrated that a decrease in myocardial ECV correlated with hematologic response. This decrease in ECV was also linked to better long-term survival. However, this positive change in the heart appeared slowly: significant ECV reductions were typically seen only after 24 months of therapy. Conversely, early disease progression, defined as an ECV increase of ≥0.05 at 6 months, was associated with a poor prognosis and may signal the need for treatment adjustment [84]. Therapeutic response may manifest differently across cardiac chambers. A prospective multicenter study found that at 12 months post-treatment, patients achieving a very good partial hematologic response (≥VGPR) exhibited a more pronounced decline in RV-ECV compared with LV-ECV, suggesting that RV improvement may precede LV response [85]. Beyond the heart, ECV mapping allows for simultaneous multi-organ assessment. A large-scale study concurrently evaluated the ECV in the heart, liver, and spleen, using SAP scintigraphy as a reference standard. Changes in the organ-specific ECV pre- and post-treatment strongly correlated with changes in SAP scintigraphy activity. Notably, ECV improvement in the liver and spleen became detectable as early as 6 months, preceding observable improvement in the heart (12 months). The baseline myocardial/hepatic ECV and the magnitude of ECV change at 6 months served as independent predictors of mortality [86].

In addition to the ECV, native T1 and T2 values serve as quantifiable treatment monitoring parameters. Early studies suggest that post-therapy reductions in T1 and T2 are associated with a lower risk of mortality and positive organ response [87,88]. However, these parameters should be interpreted in conjunction with biochemical markers when assessing organ response. Furthermore, their routine clinical utility remains to be validated through large-scale, prospective, multicenter studies.

### 3.4. Key Points

CMR provides key parameters (LGE, native T1 and T2, the ECV) for tissue characterization. LGE patterns aid differentiation from other cardiomyopathies, while native T1 and ECV quantify the amyloid burden, enabling early detection. The ECV is a powerful prognostic biomarker; T2 mapping and perfusion imaging offer additional prognostic insights. Serial ECV assessment tracks treatment response by measuring amyloid clearance, correlating with survival outcomes. Multi-organ ECVs permit comprehensive response evaluation. Native T1 and T2 show promise as monitoring tools.

## 4. Nuclear Medicine Imaging

**Figure 2:**
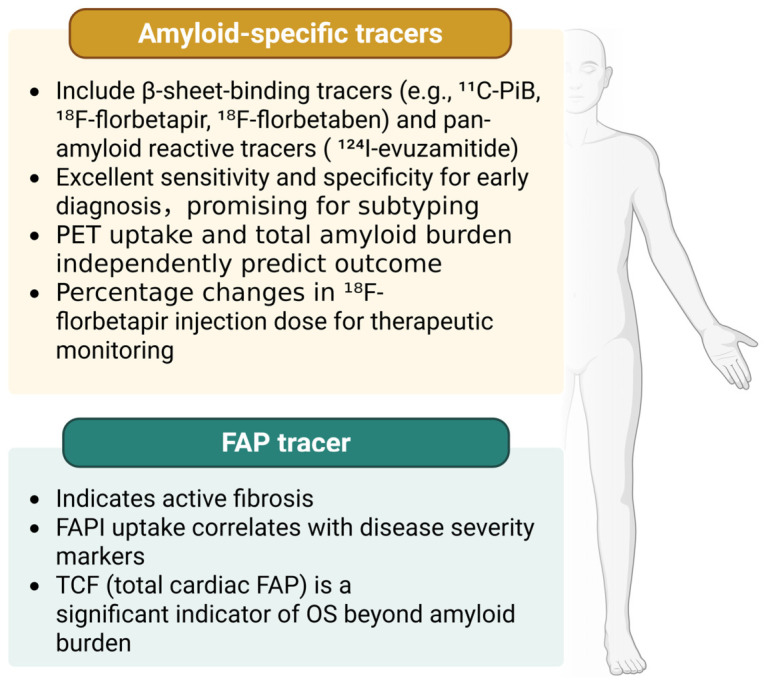
Advances in PET imaging of AL amyloidosis. PET, positron emission tomography; FAP, fibroblast activation protein; FAPI, fibroblast activation protein inhibitor; OS, overall survival. Created in BioRender. Qiu, M. (2026). https://BioRender.com/1cbwjd3 (accessed on 26 May 2026).

Nuclear medicine imaging utilizes radionuclide tracers to directly target amyloid deposits. This provides a unique, non-invasive method of quantifying the amyloid burden at a molecular level and complements anatomical imaging techniques (Figure 2). Early techniques, such as serum amyloid P (SAP) component scintigraphy, can detect various amyloid types but are limited by their lack of cardiac uptake and availability [89,90]. The development of PET-targeted probes has revolutionized the field of AL amyloidosis research. β-sheet-binding tracers (e.g., ^11^C-PiB, ^18^F-florbetapir, ^18^F-florbetaben) visualize the common fibril structure, typically showing higher uptake in AL than in ATTR and other types of amyloidosis. The short half-life of ^11^C-PiB limits its use, whereas ^18^F-labeled analogs are more practical. The pan-amyloid reactive tracer ^124^I-evuzamitide binds to a universal site on amyloid fibrils, enabling assessment of the total amyloid burden across all types, aided by its long half-life. In addition to tracers that bind amyloid fibrils, PET agents reflecting molecular pathological changes—exemplified by fibroblast activation protein inhibitor (FAPI)—are being applied in AL-CA management.

Important regulatory and practical limitations should be recognized. The 18F-labeled beta-amyloid tracers florbetapir, florbetaben, and flutemetamol are approved for beta-amyloid imaging in Alzheimer’s disease, not for cardiac amyloidosis; their cardiac use is therefore off-label and investigational in most settings.

### 4.1. Diagnosis

Amyloid-specific PET provides a highly sensitive and specific approach for diagnosing CA, crucial for both differentiation from phenotypically similar conditions and amyloid subtyping.

The β-sheet-binding tracer ^11^C-PiB exemplifies this high performance. Rosengren et al. reported sensitivities and specificities exceeding 93% for detecting CA, even in the absence of LV wall thickening. When used with semi-quantitative indices (SUVR, RI), ^11^C-PiB distinguished AL-CA from ATTR-CA with 100% accuracy, reflecting its higher avidity for AL-type fibrils [91]. Combining complementary tracers with computational models represents a promising frontier. Hong et al. integrated ^11^C-PiB PET and ^99^ᵐTc-DPD SPECT, leveraging their respective affinities for AL and ATTR subtypes [92]. A machine learning model applied to these multi-modal data achieved an AUC of 0.94 for subtype discrimination, while a principle-based model yielded AUCs of 0.95 for ATTR-CA and 0.88 for AL-CA, demonstrating the potential of integrated diagnostic platforms.

Differential kinetics were corroborated by studies with ^18^F-florbetaben, which showed sustained myocardial retention in AL-CA compared with rapid washout in ATTR-CA and other myocardiopathies. It enabled subtype stratification (AL > AA > ATTR) [93,94]. Beyond the heart, PET enables systemic burden quantification. ^18^F-florbetapir PET-CT effectively detects active AL amyloidosis in extracardiac organs, with higher detection rates in the parotid glands, tongue, and lungs when compared with the international consensus definition of organ involvement [95].

The pan-amyloid tracer ^124^I-evuzamitide exhibited 93.6% sensitivity, and its whole-body uptake correlated with clinical severity. In AL amyloidosis patients, myocardial uptake (SUVRmean) correlated with NT-proBNP levels [96].

A meta-analysis confirmed the high diagnostic performance of PET in systemic amyloidosis, reporting the highest sensitivity for ^124^I-evuzamitide (98%) and the highest specificity for ^11^C-PiB (84%). PET was notably more effective in detecting cardiac involvement in AL versus ATTR amyloidosis [97].

### 4.2. Prognosis

PET is increasingly recognized as a powerful tool for risk stratification and outcome prediction in AL-CA, offering unique insights into the amyloid burden and the associated active pathophysiology.

The degree of myocardial amyloid deposition, as quantified via PET, carries significant prognostic information. ^11^C-PiB myocardial uptake has been established as an independent predictor of major clinical outcomes, including all-cause mortality and heart transplantation [98]. This finding was validated in a study showing that ^11^C-PiB uptake correlated with one-year mortality, with scan-positive patients having a worse prognosis across high-risk biomarker subgroups [99].

Recent work emphasizes assessing the amyloid load in specific chambers. A study demonstrated that ^18^F-florbetapir PET/CT could detect RV amyloid deposition before structural or functional changes, and that this elevated RV burden independently predicted RV dysfunction and major adverse cardiac events [100]. Similarly, a study using ^18^F-florbetaben and the metric of total amyloid burden (TAB) found that late-phase LV TAB ≥ 273 cm^3^ and RV TAB ≥ 135 cm^3^ independently predicted 18- and 24-month all-cause mortality, even after adjustments for established risk factors [101]. The independent prognostic value of LV amyloid burden alone, however, is debated. While supported by the TAB data, another study reported that the predictive value of the LV burden for major adverse cardiac events was not independent of the Mayo stage and was mediated by NT-proBNP [102]. These discrepancies likely arise from methodological differences, underscoring the need for standardization.

Beyond the amyloid load, imaging myocardial fibrosis via fibroblast activation protein inhibitor (FAPI) PET-CT offers a novel prognostic dimension. ^68^Ga-FAPI uptake correlates with disease severity markers in AL-CA [103]. Crucially, a prospective dual-tracer (^18^F-florbetapir/^68^Ga-FAPI-04) study revealed that the extent of myocardial fibroblast activation (quantified as total cardiac FAP) was a stronger determinant of OS than the amyloid burden itself, especially in advanced-stage patients, highlighting fibrosis as a key driver of outcomes [104].

Integrating multi-tracer PET data with machine learning represents a forward-looking approach. One study developed a model that identified key imaging signatures for predicting survival. When patients were stratified using the model’s predictions, a significant difference in overall mortality was observed (adjusted hazard ratio: 2.43). This demonstrates the potential of such computational models to unify diagnosis, subtyping, and risk stratification [92]. The generalizability of such models awaits confirmation in larger, external cohorts.

### 4.3. Monitoring Therapy Response

Molecular imaging with amyloid-specific PET tracers provides a quantitative approach for monitoring treatment response in AL amyloidosis, offering insights into dynamic changes in organ-specific amyloid burden. A prospective study by Law et al. evaluated the systemic amyloid burden using ^18^F-florbetapir PET/CT, quantified as the percentage of injected dose (%ID), and compared it with the CMR-derived ECV in AL patients. In patients with cardiac involvement (AL-CA), a significant reduction in myocardial %ID was observed as early as 6 months post-treatment, with a continued decline at 12 months. This reduction correlated moderately with changes in NT-proBNP. In contrast, the ECV showed no significant change. Notably, no significant change in %ID was seen in AL amyloidosis patients without cardiomyopathy, underscoring the specificity of this parameter for tracking cardiac-specific treatment response [105]. A four-year longitudinal case study used sequential ^124^I-evuzamitide PET/CT scans in a treated AL amyloidosis patient. It showed marked reductions in tracer uptake in the liver, spleen, and bone marrow compared with the heart and kidneys, supporting the feasibility of serial monitoring to track organ-specific treatment response [106]. These findings collectively highlight the potential of quantitative PET metrics to serve as sensitive, organ-specific biomarkers for evaluating treatment efficacy in AL amyloidosis.

### 4.4. Key Points

Nuclear molecular imaging offers a unique approach for evaluating systemic AL amyloidosis by targeting amyloid fibrils or associated pathological processes (Table 1). PET tracers such as ^11^C-PiB and ^18^F-florbetapir enable highly sensitive and specific detection of cardiac and systemic amyloid deposition, while also allowing promising differentiation between AL and ATTR subtypes. Furthermore, quantitative assessments of amyloid burden and fibrotic activity (e.g., using ^68^Ga-FAPI) provide significant prognostic value, and serial PET imaging permits dynamic monitoring of treatment response. However, most PET applications remain investigational because regulatory indications, tracer availability, quantification standards, and external validation are incomplete. AI integration with multi-tracer data analysis is promising, but, at present, it should be viewed as hypothesis-generating and supportive rather than clinically definitive.

## 5. Conclusions

In the non-invasive evaluation of AL amyloidosis, each imaging modality offers distinct advantages and inherent limitations (Table 2). Echocardiography remains the first-line screening and follow-up tool, CMR provides the strongest tissue characterization framework, and nuclear molecular imaging offers a promising but still specialized approach to amyloid burden quantification. Multimodality imaging should be integrated with hematologic testing, tissue confirmation, amyloid typing, biomarkers, and clinical assessment, rather than used in isolation. AI and radiomics may improve efficiency and reproducibility, but most models still require external validation, interpretability assessment, and regulatory implementation before routine use (Table 3).

**Figure 3:**
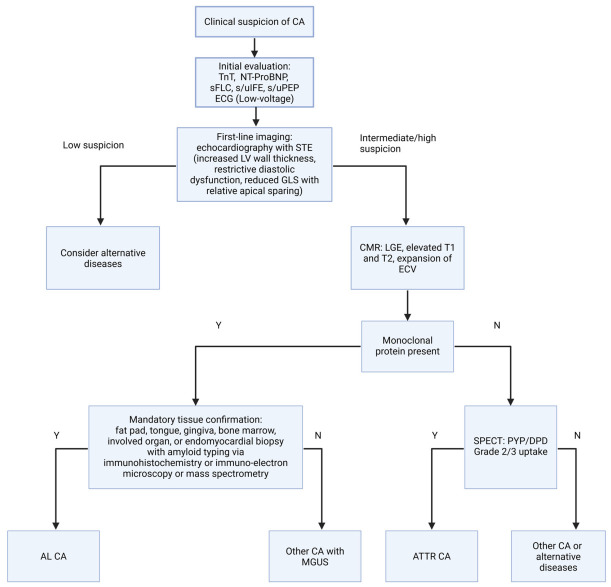
Practical diagnostic algorithm for suspected cardiac involvement in light chain amyloidosis. CA, cardiac amyloidosis; TnT, troponin T; sFLC, serum free light chain; s/u IFE, serum/urine immunofixation electrophoresis; s/u PEP, serum/urine protein electrophoresis; ECG, electrocardiography; STE, speckle-tracking echocardiography; LV, left ventricular; GLS, global longitudinal strain; CMR, cardiac magnetic resonance; LGE, late gadolinium enhancement; ECV, extracellular volume fraction; AL, light chain; ATTR, transthyretin amyloidosis; SPECT, single-photon emission computed tomography; PYP, technetium-99m pyrophosphate; DPD, technetium-99m 3,3-diphosphono-1,2-propanodicarboxylic acid; Y, yes; N, no. Created in BioRender. Qiu, M. (2026). https://BioRender.com/noyc6r3 (accessed on 26 May 2026).

It is imperative to reinforce a fundamental diagnostic principle distinguishing AL from ATTR cardiac amyloidosis (Figure 3). Unlike ATTR, where Grade 2 or 3 bone scintigraphy in the absence of a monoclonal protein permits a non-invasive diagnosis, AL amyloidosis invariably demands histopathological confirmation demonstrating light chain amyloid deposition in an affected tissue or organ. This distinction underscores that while imaging guides suspicion and disease burden monitoring, it cannot replace the mandatory tissue diagnosis required to initiate targeted anti-plasma cell therapy.

Finally, multimodal imaging has propelled the field beyond mere anatomical assessment into an era of precision medicine, becoming pivotal for diagnosis, risk stratification, and therapeutic response monitoring. However, the generalizability of these advanced imaging biomarkers, particularly novel quantitative metrics and AI-driven algorithms, necessitates further validation through large-scale, prospective, multicenter studies. Looking ahead, synergy between effective disease-modifying therapies and personalized imaging phenotypes will be instrumental in improving patient outcomes through early detection and dynamic, individualized management.

## Figures and Tables

**Table 1:** PET radiotracers in AL amyloidosis.

Tracer	Molecular Target	Main Applications	Strengths	Limitations	Level of Evidence
11C-PiB	Beta-sheet amyloid fibrils	Cardiac amyloid detection; AL/ATTR differentiation; prognostic assessment	High diagnostic performance; useful subtype signal; quantitative uptake metrics	Short half-life requires on-site cyclotron; limited availability	Promising specialized-center tool; evidence from clinical cohorts; still not routine
18F-florbetapir	Beta-sheet amyloid fibrils	Cardiac and multi-organ amyloid detection; prognosis; treatment response monitoring	Long half-life; whole-body assessment; quantitative burden metrics	Approved for Alzheimer’s disease, off-label cardiac use	Investigational for AL amyloidosis; requires multicenter validation
18F-florbetaben	Beta-sheet amyloid fibrils	Differential diagnosis; total amyloid burden; prognosis	Long half-life; delayed retention may help distinguish AL from ATTR in selected studies	Approved for Alzheimer’s disease, off-label cardiac use	Promising but not routine; cohort-level evidence
18F-flutemetamol	Beta-sheet amyloid fibrils	Potential cardiac amyloid detection	Long half-life	Less cardiac amyloidosis evidence	Research-stage for AL cardiac applications
124I-evuzamitide	Pan-amyloid fibrils	Whole-body amyloid burden assessment; serial organ response imaging	Targets multiple amyloid types; long half-life; high sensitivity	Limited availability; early-phase evidence	Investigational; early clinical studies
68Ga-FAPI	Fibroblast activation proteins	Assessment of fibroblast activation; prognostic stratification in AL-CA	May capture active fibrotic response beyond amyloid burden	Does not directly show amyloid fibrils; specificity and response criteria require validation	Research-stage; promising prognostic biomarker

AL: light chain cardiac amyloidosis; ATTR: transthyretin amyloidosis; CA: cardiac amyloidosis; FAPI: fibroblast activation protein inhibitor.

**Table 2:** Imaging advances in light chain amyloidosis.

Modality	Key Advantages	Key Limitations	Clinical Readiness
Echocardiography	Widely available and cost-effective, making it the first-line tool for screening and follow-up. Speckle-tracking echocardiography sensitively detects subclinical dysfunction. GLS serves as a core parameter with robust evidence for prognostic stratification and therapy monitoring.	Individual parameters (e.g., wall thickness) have limited specificity. Operator-dependent. The threshold for GLS is not yet standardized.	Full clinical readiness
Magnetic resonance imaging	Establishes the reference standard for tissue characterization, providing comprehensive structural and tissue data. The ECV is the strongest biomarker for quantifying amyloid burden and predicting prognosis. Enables multi-organ assessment (heart, liver, spleen) for comprehensive treatment response evaluation.	High cost and limited accessibility. Contraindicated in patients with certain metallic implants or severe renal impairment (when contrast is used). Treatment response (e.g., ECV change) is often delayed, typically observed after 12–24 months.	Conditional clinical readiness
Nuclear medicine	Molecular imaging allows direct, specific targeting of amyloid deposits or pathological changes (e.g., fibroblast activation). Promising for subtyping amyloidosis (e.g., differentiating AL from ATTR). Whole-body assessment capability for detecting extracardiac involvement. May detect changes in amyloid burden earlier following treatment initiation.	Limited availability, high cost, and radiation exposure. Regulatory limitations. Lack of standardized quantification methods and response criteria. Short half-life of some tracers (e.g., ^11^C-PiB) restricts clinical use.	Limited clinical readiness

AL: light chain cardiac amyloidosis; ATTR: transthyretin amyloidosis; ECV: extracellular volume; GLS: global longitudinal strain.

**Table 3:** Artificial intelligence applications in imaging of AL amyloidosis.

Imaging Technique	AI/Radiomics Application	Reported Use	Current Level of Evidence	Degree of Clinical Implementation
Echocardiography + ECG	Machine learning or deep learning screening models	Detection of CA and differentiation from phenocopies	Retrospective or selected cohorts; external validation limited	Emerging decision support tool, not for stand-alone diagnosis
Echocardiographic video	Deep learning from cine/video data	Automated CA detection and referral support	Promising performance in development datasets	Research stage; requires prospective workflow validation
CMR LGE/cine imaging	Deep learning detection and subtyping	CA detection; AL/ATTR/HCM differentiation	High AUCs reported in recent studies, but scanner and protocol heterogeneity remain important	Promising specialized-center support
CMR LGE radiomics	Radiomic signatures for prognosis	All-cause mortality prediction and incremental risk stratification	Multicenter retrospective evidence is emerging	Not routine; needs reproducibility and calibration studies
PET/SPECT multimodal imaging	Computational integration of tracer uptake patterns	Subtype discrimination and survival modeling	Early evidence using combined 11C-PiB PET/CT and 99mTc-DPD scintigraphy	Research stage

AI: artificial intelligence; ECG: electrocardiography; CA: cardiac amyloidosis; CMR: cardiac magnetic resonance; LGE: late gadolinium enhancement; AL: light chain cardiac amyloidosis; ATTR: transthyretin amyloidosis; HCM: hypertrophic cardiomyopathy; AUC: area under the receiver operating characteristic curve; PET: positron emission tomography.

## Data Availability

No new data were created or analyzed in this study. Data sharing is not applicable to this article.

## Abbreviations

The following abbreviations are used in this manuscript:

AL	Light chain
ATTR	Amyloid transthyretin
NT-proBNP	N-terminal pro-brain natriuretic peptide
OS	Overall survival
MRI	Magnetic resonance imaging
CA	Cardiac amyloidosis
STE	Speckle-tracking echocardiography
GLS	Global longitudinal strain
ECG	Electrocardiography
HCM	Hypertrophic cardiomyopathy
HHD	Hypertensive heart disease
AUC	Area under the receiver operating characteristic curve
RAS	Relative apical sparing
RV	Right ventricular
LA	Left atrial
3DSTE	Three-dimensional STE
HFpEF	Heart failure with preserved ejection fraction
RWT	Relative wall thickness
TAPSE	Tricuspid annular plane systolic excursion
EFSR	Ejection fraction/peak systolic global longitudinal strain ratio
FAC	Fractional area change
AI	Artificial intelligence
LACI	Left atrioventricular coupling index
PA	Pulmonary arterial
MW	Myocardial work
GWI	Global myocardial work index
GCW	Global constructive work
CMR	Cardiac magnetic resonance
LGE	Late gadolinium enhancement
ECV	Extracellular volume fraction
MDI	Myocardial diffusion index
MBF	Myocardial blood flow
VGPR	Very good partial hematologic response
SAP	Serum amyloid P
FAPI	Fibroblast activation protein inhibitor
AA	Amyloid A
TAB	Total amyloid burden
ID	Injected dose
